# Survey of Skin-to-Skin Contact with Obstetrics and Pediatric Nurses

**DOI:** 10.3390/nursrep12010002

**Published:** 2022-01-13

**Authors:** Wedad M. Almutairi

**Affiliations:** Maternity and Child Department, Faculty of Nursing, King Abdulaziz University, Jeddah 21551, Saudi Arabia; walmutairi@kau.edu.sa

**Keywords:** skin-to-skin contact, nurses, knowledge, beliefs/attitudes, implementation

## Abstract

Skin-to-skin, or chest-to-chest, contact (SSC) between newborns and their mothers is known as kangaroo mother care. The physiological and psychological benefits of SSC for infants and mothers are well established. The World Health Organization (WHO) recommends practicing SSC for term and preterm newborns. However, in Saudi Arabia, SSC is not practiced as widely as recommended. There is insufficient evidence of the nurses’ knowledge and attitudes regarding SSC in Saudi Arabia. The aims of this study were to describe and determine relationships between knowledge, education, beliefs/attitudes, and the implementation of SSC in Jeddah, Saudi Arabia. Thank You for Your Time and Kind Suggestion Methods: Cross-sectional descriptive data were collected from 40 nurses using an English-language version of a knowledge, beliefs/attitudes, education, and implementation questionnaire used by others. Results: The mean age of the nurses was 42.4 years (SD = 3.2), with a mean experience of 12 years (SD = 2.1). The mean total score of SSC knowledge was 13.6 (SD = 2.3), the mean of total score of attitudes/beliefs was 12.3 (SD = 3.1), the SSC education mean score was 17.1 (SD = 3.4), and the SSC implementation mean score was 17.0 (SD = 4.1). In total, 55% of the nurses were not sure of the impact of SSC on brain development in neonates, 45% could not interpret infants’ responses during SSC; 67% disagreed that it was the nurses’ responsibility to facilitate SSC, 37.5% were not aware of SSC guidelines, and 47% of the nurses had not received any continuing education on SSC in their units. Pearson correlations revealed a significant association between SSC implementation and nurses’ knowledge level (r = 0.297, *p* = 0.031), education (r = 0.85, *p* = 0.015), and beliefs (r = 0.31, *p* = 0.024). Conclusions: Once nurses have improved their knowledge, education, and beliefs/attitudes, SSC implementation may concomitantly increase. A continuing education program and clear guidelines are needed to promote SSC adoption in practice.

## 1. Introduction

Skin-to-skin contact (SSC) between mothers and newborns, or kangaroo mother care (KMC), is defined as “placing the naked baby prone on the mother’s bare chest at birth or soon afterwards” [1]. The World Health Organization (WHO) defined KMC as early, continued, and prolonged SSC with exclusive breastfeeding [2]. Routine implementation of SSC is encouraged because its beneficial effects on pre- and full-term infants and their mothers have been extensively studied and documented: physiological stability, improved cardiorespiratory function, reduced hypoglycemia, better thermoregulation, less pain, earlier breastfeeding initiation, and longer duration and exclusivity of breastfeeding [1,3,4,5,6] as well as better sleep and brain maturation [1,6,7,8]. Moreover, SSC supports the physiological and psychological status of the mothers. SSC enhances maternal emotional well-being, with mothers experiencing less stress and an increased sense of competence. SSC also reduces postpartum depression symptoms [9,10] and has a role in preventing postpartum hemorrhage by decreasing the duration of the third stage of labor and reducing postpartum blood loss [11,12,13]. However, global SSC implementation is less than routine, causing researchers to determine why [14].

There have been several studies of nurses’ knowledge, beliefs/attitudes, education, and implementation of SSC. National studies conducted in the USA began in 2002 with Engler et al.’s study showing that 82% of 637 responding hospitals offered SSC to mothers who requested it and that the nurses’ knowledge of the benefits of SSC was poor, in which inadequate knowledge negatively impacted the nurses’ attitudes toward SSC [15]. In the same year, Franck et al. (2002) conducted a national survey of 215 randomly selected NICUs and learned that of the units in which nurses had been educated about SSC, 45% practiced SSC with intubated infants, 73% with extubated infants, 68% with fathers providing SSC, and 2% allowing siblings to provide SSC [16]. In 2017, Vittner et al. (2017) surveyed 79 perinatal nurses’ knowledge, attitudes, and practice of SSC and found that knowledge of SSC benefits was limited and attitudes played a role in the low number of units in which SSC was practiced regularly [17]. A recent national survey in China showed that SSC is uncommon in China, and that a major barrier to SSC was physician, nurse, and/or parent reluctance; reluctance in nurses was due to nurses not knowing about SSC’s benefits and each nurse having to care for five NICU infants each day [18]. Furthermore, a recent web-based questionnaire study conducted in Saudi Arabia, which surveyed 209 NICU nurses to assess their knowledge and competency levels and identify the barriers to practice, found that most respondents perceived KMC as promoting mother–infant bonding and enhancing successful breastfeeding, but they were uncertain of the KMC application. The authors recommended the provision of education programs to facilitate the recommended implementation of KMC in Riyadh [19]. A cross-sectional survey conducted in two public hospitals and one private hospital, which aimed to assess the attitudes and practices of kangaroo mother care by maternity nurses, revealed an association between the type of degree held by nurses and their attitudes. Nurses who held a master’s degree had more positive attitudes toward KMC compared to those with bachelor degrees. Continuing education is essential for practicing KMC as recommended [20]. In their study, Pados et al. encouraged the use of SSC to minimize stress in neonates and stressed that nurses with a positive attitude toward SSC had a “key role in maximizing SSC’s use” [21]. Knowledge and attitudes are crucial factors in enabling nurses to implement SSC practices.

To facilitate SSC practice in Saudi Arabia, the knowledge and attitudes of the nurses across the country must be studied; however, to the best of our knowledge, no studies on SSC practices have been conducted in the city of Jeddah, which represents the western region of the kingdom.

Objectives: The objectives of this study were to describe the nurses’ knowledge, education, beliefs/attitudes, and implementation of SSC, and to determine any relationships between them.

## 2. Materials and Methods

Design: A cross-sectional correlational descriptive study was used to describe the nurses’ knowledge, education, beliefs/attitudes, and implementation of SSC and to determine the relationships between them.

Setting: All nurses were recruited from an obstetric and pediatric department (NICU), labor room, and postpartum care unit at a university hospital. Period of recruitment was from 20 March 2018 to 5 April 2018.

Participants: The participant population included nurses working in obstetric and pediatric units, which are units in a hospital where SSC between mothers and newborns is applied. A convenience sampling method was used to gain access to study participants. Data collectors were responsible for handing the surveys to nurses who agreed to fill in the survey and then collecting the surveys from them when they had completed it; alternatively, they could leave the completed survey in the head nurse’s office in their unit after completion. Consent was implied if the participants agreed to complete the survey and handed it back to the data collectors or head nurse of the unit. All nurses were English speakers.

Variables and Measurement: The study variables were SSC knowledge, attitudes and beliefs, SSC education, and SSC implementation. The predictor variables were SSC knowledge, attitudes and beliefs, and SSC education, and the outcome variable was SSC implementation. The tool was a shortened (20 items) version of Engler et al.’s 2002 SSC survey of nurses’ knowledge, attitudes, and practices of SSC (Engler et al., 2002) by Vittner et al. The 20 items were divided into four dimensions: SSC Knowledge (five items), Attitudes and Beliefs (four items), SSC Education (five items), and SSC Implementation (six items). The five-point Likert scale responses ranged from Strongly Disagree (SD) to Disagree (D), to Neither Agree nor Disagree (N), to Agree (A), to Strongly Agree (SA). The Likert data were treated as continuous data in which 1 = SD, 2 = D, 3 = N, 4 = A, and 5 = SA. Thus, the possible range of scores for Knowledge was 5–25, for Attitudes and Beliefs 4–20, for Education 5–25, and for Implementation 6–30. The tool’s construct validity was established by conducting a principal component analysis, and the reliability of the subscales ranged between 0.79 and 0.90 [15,17].

Ethical Consideration: The study was reviewed and approved by the biomedical ethics research committee at the university hospital. Reference# 131-18.

Bias: We controlled the non-response bias by choosing a short, simple survey that helped us to increase the participants’ response rate in a tertiary setting where nurses have a heavy workload. We chose a cross-sectional design, which allowed us to collect all responses at once to control any events that might affect the nurses’ responses. We controlled data collection bias by recruiting data collectors who were responsible for collecting the data and entering the data into the Statistical Package for Social Sciences (SPSS) program, then checking the entry with research assistance to avoid any potential bias from the researcher’s knowledge.

Study size: A total of 44 nurses working in obstetric and pediatric units in the selected setting. We calculated our sample size using power analysis with 95 CI, margin error 5%, and population proportion of 50.

Quantitative variables and statistical methods The statistical analysis involved descriptive statistics and Pearson product–moment correlations using SPSS, version 16.0. Frequency and percentage were used to describe the distribution of responses across the Likert scale for each item, and the score for each item was reported as an interval number. The mean ± SD was used to describe Likert data treated as continuous data. The Pearson correlation was used to identify the relationship between the study variables. *p*-values of <0.05 were considered significant. The dimension scores (total of scores for all items in each dimension) were used for the correlations.

## 3. Results

Participants/descriptive data: A total of 40 nurses responded completely: 12 were from NICU, 13 from the labor room, and 15 from the postpartum care unit. In total, 50% of the nurses were Pilipino and the remaining were Indian, Saudi, and Egyptian, representing 30%, 15%, and 5%, respectively. All nurses were female with a mean age of 42.4 (SD = 3.2) years old, and average years of experience of 12 (SD = 2.1). Most participants held a Bachelor’s degree in Nursing (75%) and 25% had a diploma.

Main results: The number and percentage *of* Likert-based responses on the nurses’ *knowledge* of SSC are shown in Table 1; nurses’ attitudes/beliefs toward SSC are shown in Table 2; SSC education is shown in Table 3; and nurses’ perceptions toward SSC implementation are shown in Table 4. The *mean of* the total *score* of the SSC knowledge dimension was 13.6 (SD = 2.3), the mean of the total score of attitudes/beliefs was 12.3 (SD = 3.1), SSC education mean score was 17.1 (SD = 3.4), and SSC implementation mean score was 17.0 (SD = 4.1).

Table 1 shows that 45% of the participants disagreed that they were not confident in their ability to interpret infant response during SSC. Similarly, 40% disagreed that they lacked SSC in the neonatal period, and that it therefore has long-term adverse effects. Moreover, 37.5% of the nurses were not sure about whether the SSC could reduce the risk of impaired brain development in neonates, whereas 55% disagreed that SSC changes brain growth in the neonate. The mean total score of 13.6 (SD = 2.3) indicates a low knowledge of SSC.

Table 2 shows that 67% of the nurses disagreed that SSC intervention should be advocated by the nurses, 52% [21] disagreed that discomfort from minor procedures such as gavage tube placement and oral suctioning could be minimized with SSC, and 50% did not feel confident in their skills to safely facilitate SSC, while 47% agreed that SSC is effective in reducing the risks associated with physical separation of the neonate and their mothers. The total mean score of the nurse’ attitudes/beliefs toward SSC was 12.3 (SD = 3.1), indicating relatively positive attitudes.

Table 3 shows that 47% of the nurses disagreed that their units provided any continuing education about SSC; 37.5% were not aware of SSC guidelines or protocols in their units; and 37.5% had not attended any educational activities including SSC in the last five years. However, 47.5% used assessment tools for SSC in their units and 45% used accurate measurements of infant responses during SSC (Table 3). The total mean score for SSC education was 17.1 (SD = 3.4), which indicates that nurses had a moderate amount of continuing education about SSC in their units.

Table 4 shows that most of the nurses (42%) disagreed that the SSC guidelines/protocols were clear and 37.5% disagreed that physicians were willing to implement new evidence-based application of SSC in their unit. Furthermore, 32.5% did not receive any training regarding SSC during the orientation. On the other hand, 35% of the nurses agreed that they received adequate education during the unit orientation and also agreed that their units used SSC regularly (42.5). Moreover, most of the nurses (35%) agreed that they used SSC with eligible neonates (Table 4). The total score mean of SSC implementation was 17.0 (SD = 4.1), indicating moderate implementation of SSC in the setting.

To determine relationships between nurses’ knowledge, education, beliefs/attitudes, and implementation of SSC, Pearson correlation findings revealed a significant positive association between implementation of SSC and the level of nurse’s knowledge (r = 0.297, *p*-value = 0.03), SSC implementation and nurse’s attitudes (r = 0.316, *p* = 0.02), and SSC implementation and nurse’s education (r = 0.846, *p* = 0.00). A significant association was found between nurse’s knowledge, attitudes, and education (knowledge and attitudes significantly associated (r = 0.4, *p* = 0.006)). Moreover, a significant association was found between nurse’s attitudes/beliefs and their education (r = 0.34, *p* = 0.02), as shown in Table 5.

## 4. Discussion

Key results: The findings showed that nurses had a moderate level of knowledge, positive attitudes/beliefs, moderate education, and moderate implementation levels. The findings also revealed a significant association between nurse’s knowledge, attitudes/beliefs, SSC education about SSC, and nurses’ perceptions toward SSC implementation in a tertiary hospital.

Limitations: There are several limitations to this study. A relatively small sample size of participants was used, which was due to the selection being limited to pediatric and obstetric units, which have a smaller number of staff compared to other units. Furthermore, the participants were selected from only one setting in the city of Jeddah, which will affect the generalizability of the findings. Finally, based on the study design, a causal relationship cannot be inferred. However, the results are consistent with other studies conducted in different cities in Saudi Arabia.

Interpretation of the nurses’ SSC Knowledge: The results indicate a very low knowledge level of SSC in obstetric and pediatric unit nurses. Nurses demonstrated uncertainty about their information regarding infants’ physiological and psychological responses during SSC and how they assessed or interpreted these responses. Moreover, most of them were not aware of the neurological benefits of SSC for infants’ brain maturation. The study results are comparable to Al-Shehri and Binmanee in Riyadh, Saudi Arabia, who found that nurses were uncertain about physiological responses for low-weight infants [19]. The study results are also comparable to Mallet et al.’s findings that showed a low level of knowledge of the effects of KMC on infant’s sleep, breastfeeding, and pain [22]. The findings are comparable to a survey study with interviews that investigated the role of neonatal nurses in adopting KMC innovation. The results show that there is ambiguity about SSC knowledge regarding the effects of SSC on breastfeeding, safety of SSC, and suitability of the infants to have SSC [23]. Our findings are also comparable to a national survey study conducted in China with neonatal nurses, which showed a relatively low level of nurses’ knowledge about SSC [18]. On the other hand, our findings were in contrast to a national survey study’s findings conducted in the United States in 2002, where the nurses demonstrated a high level of SSC knowledge [15]. This variation between nurses in Saudi Arabia and the USA might be explained by the efforts in the USA to facilitate and support the implementation of SSC in all health settings and support all KMC initiations through a non-profit organization such as the United States Institute of Kangaroo Mother Care (USIKC), which offers a certification program every year to enhance and update nurses’ SSC knowledge and skills.

Interpretation of nurses’ attitudes/beliefs toward SSC: Nurses have a relatively positive attitude toward SSC benefits for the newborn, which is comparable to several studies that showed a positive attitude toward SSC benefits in mother–infant bonding [17,20,22,24]. Although the overall score of the attitudes dimension was positive, a majority of the nurses disagreed with the statement about the effects of SSC on reducing the discomfort of minor procedures, which is in agreement with Mallet et al.’s results [22]. A majority of the nurses disagreed with the statement that the nurse’s responsibility is to be an advocate for skin-to-skin holding for neonates in their care and facilitation of SSC practices, which contradicts the findings of other studies by Vittner et al., which revealed the nurse’s certainty of their responsibility to advocate and facilitate SSC implementation [17] and Solomon’s findings, which showed that 66.7% of the nurses agreed that they should always facilitate SSC [24]. Moreover, most nurses disagreed with the statement, “I feel confident in my skills to safely facilitate skin-to-skin holding with neonates”, which reflects the low level of nurse’s confidence in facilitating SSC safely. This finding is comparable to the national survey in China in which nurses chose safety concerns as one of the barriers to implementing SSC [18].

Interpretation of Nurse’s SSC Education: Nurses showed a moderate level of education, awareness of guidelines in their units, regular assessment, the measurement of SSC responses, and attending educational courses or conferences on SSC. However, most of the nurses’ responses indicated a lack of SSC continuing education in their units, which is comparable to findings of a perinatal nurses survey, which showed that most of the nurses did not have continuing-education services in their units regarding SSC practices [17]. A lack of education and training is one of the main barriers to SSC implementation [18,19,22,24,25,26,27].

Interpretation of nurses’ perceptions toward SSC implementation. Nurses demonstrated moderate implementation of SSC in their units. Obstetric and pediatric units use SSC regularly with eligible neonates with good management of SSC practices, but there are no clear guidelines and protocols updated with current evidence. This finding is in agreement with several previous studies conducted worldwide and clearly shows the importance of having clear guidelines and protocols to facilitate SSC adoption and implementation of the practices [15,17,18,19,24,28]. Moreover, most of the nurses disagreed about the physicians’ willingness to use SSC, which is also one of the barriers to the adoption of SSC in practice [18].

The association of nurses’ knowledge, education, and attitudes/beliefs with SSC implementation aligned with several previous studies, which found that confidence increases when the level of knowledge increases [29] and positive attitudes toward SSC appear [20]. Education also has an important role in facilitating the faster adoption of SSC practices; when the nurses or health care providers have continuing and updated education regarding SSC practices, the implementation of SSC will also increase [20,27,28].

There are conflicting responses regarding SSC implementation, while guidelines and protocols were unclear, not enough training and education was received, and the physician did not support SSC in their unit’s SSC practices regularly with eligible neonates. This might be explained by the Saudi Ministry of Health’s initiation toward practicing SSC through a baby-friendly hospital policy that encourages hospital representatives to implement SSC in each hospital, but without the complete application of the policy by setting up a training and education plan for the staff and providing a clearly written policy with details of procedures for staff to follow and make it relevant to them in each unit policy and procedure book.

Generalizability: This study’s findings cannot be generalized for all obstetric and pediatric unit nurses because the study was conducted in one setting with a very small number of nurses working there. We cannot generalize the findings because of the nature of the setting; the study setting was a university hospital, which always has an educational role that affects its services. Moreover, 50% of the participants were Pilipino, which might also affect the generalizability, because heterogenicity of the sample was not met. However, our findings are highly applicable to nursing settings characterized by the large presence of Pilipino staff and teaching hospital.

Recommendation: Based on the findings, we recommend providing staff with continuing education on the updated evidence of SSC and a training lab to support the nurses’ skills in the implementation of SSC for term/preterm and NICU newborns. This study also recommends establishing clear policy and guidelines for the use of SSC in the policy and procedure handbooks in each pediatric and obstetric unit. During the staff orientation period, we recommend using SSC workshops to enhance the nurses’ level of knowledge and review the policy and guidelines to foster SSC adoption appropriately in practice. Regarding future studies, it is recommended that a quasi-experimental study is conducted to measure the changes in knowledge level and beliefs on SSC after intensive educational courses or workshops. Furthermore, this study should be conducted with a larger sample size in multi-settings in Jeddah City.

## 5. Conclusions

Skin-to-skin contact between newborns and mothers is evidence-based practice that should be adopted optimally according to the recommendations from the WHO and other national and international organizations. Nurses’ knowledge, education, and beliefs are important facilitators for optimal use of SSC, however, until now, many studies in Saudi Arabia have shown inadequate levels of knowledge and unclear policy and guidelines in the health care setting that impede the adoption of SSC and also have negative effects on the nurses’ beliefs and attitudes. This study provides updated information regarding the nurses’ knowledge, beliefs, and education that have a direct influence on the implementation of SSC in Jeddah, Saudi Arabia.

## Figures and Tables

**Table 1:** Number and percentage of Likert-based responses on the nurses’ knowledge of SSC.

SSC Knowledge	SD	D	N	A	SA
	N (%)	N (%)	N (%)	N (%)	N (%)
1. I am confident in my ability to interpret infant responses during SSC.	4 (10%)	18 (45%)	5 (12.5%)	10 (25%)	3 (7.5%)
2. Lack of SSC in the neonatal period has long-term adverse effects.	5 (12.5%)	16 (40%)	11 (27.5%)	6 (15%)	2 (5%)
3. SSC can reduce the risk of impaired brain development in neonates.	4 (10%)	14 (35%)	15 (37.5%)	5 (12.5%)	2 (5%)
4. I feel confident with my skills in recognizing and assessing the physiological/behavioral responses of infants during SSC.	4 (10%)	16 (40%)	6 (15%)	13 (32%)	1 (2.5%)
5. SSC changes brain growth in the neonate.	2 (5%)	22 (55%)	12 (30%)	3 (7.5%)	1 (2.5%)

**Table 2:** Number and percentage of Likert-based responses on the nurses’ beliefs and attitudes toward SSC.

SSC Attitudes and Beliefs	SD	D	N	A	SA
	N (%)	N (%)	N (%)	N (%)	N (%)
6. Discomfort from minor procedures, such as gavage tube placement and oral suctioning, can be minimized with SSC.	3 (7.5%)	21 (52.5%)	6 (15%)	9 (22.5%)	1 (2.5%)
7. It is the responsibility of nurses to be an advocate for skin-to-skin holding for neonates in their care.	4 (10%)	27 (67.5%)	6 (15%)	2 (5%)	1 (2.5%)
8. SSC is effective in reducing risks associated with physical separation of neonates and their mothers.	1 (2.5%)	1 (2.5%)	5 (12.5%)	19 (47.5%)	14 (35%)
9. I feel confident in my skills to safely facilitate skin-to-skin holding with neonates.	4 (10%)	20 (50%)	5 (12.5%)	9 (22.5%)	2 (5%)

**Table 3:** Number and percentage of Likert-based responses on the nurse’s SSC education.

SSC Education	SD	D	N	A	SA
	N (%)	N (%)	N (%)	N (%)	N (%)
My unit provides continuing education regarding SSC.	2 (5%)	19 (47.5%)	3(7.5%)	9 (22.5%)	7 (17.5%)
I am aware of SSC guidelines/protocols in my unit.	2 (5%)	15 (37.5%)	0 (0%)	14 (35%)	9 (22.5%)
My unit regularly uses an assessment tool for SSC responses.	0 (0%)	8 (20%)	4 (10%)	19 (47.5%)	9 (22.5%)
The way we measure responses to SSC on my unit is an accurate measurement of infant responses.	0 (0%)	8 (20%)	6 (15%)	18 (45%)	8 (20%)
I have attended an educational course/conference/lecture that has included SSC within the last five years.	1 (2.5%)	15 (37.5%)	0 (0%)	13 (32.5%)	11 (27.5%)

**Table 4:** Number and percentage of Likert-based responses on the nurses’ perceptions of SSC implementation.

Nurses’ Perceptions of SSC Implementation	SD	D	N	A	SA
	N (%)	N (%)	N (%)	N (%)	N (%)
I feel that the provision of SSC in my unit is well managed.	0 (0%)	9 (22.5%)	8 (20%)	15 (37.5%)	8 (20%)
My unit uses skin-to-skin holding regularly.	1 (2.5%)	11 (27.5%)	3 (7.5%)	17 (42.5%)	8 (20%)
The healthcare providers in my unit practice adequate SSC with eligible neonates.	0 (0%)	11 (27.5%)	6 (15%)	14 (35%)	9 (22.5%)
Physicians are willing to use new evidence-based application of SSC in my unit.	0 (0%)	15 (37.5%)	10 (25%)	10 (25%)	5 (12.5%)
I received adequate education or training regarding SSC when I was oriented to my unit.	0 (0%)	13 (32.5%)	4 (10%)	14 (35%)	9 (22.5%)
The SSC guidelines/protocols are clear, comprehensive, and based on current research.	0 (0%)	17 (42.5%)	5 (12.5%)	12 (30%)	6 (15%)

**Table 5:** Correlation analysis.

	Knowledge	Attitudes and Beliefs	Education	Implementation
Knowledge	1	0.397 (0.006)	0.311 (0.025)	0.297 (0.031)
Attitudes and beliefs	0.397 (0.006)	1	0.342 (0.015)	0.316 (0.024)
Education	0.311 (0.025)	0.342 (0.015)	1	0.846 (0.000)
Implementation	0.297 (0.031)	0.316 (0.024)	0.846 (0.000)	1

## Data Availability

All data are included in the study.
